# Surface-Dominated Quantum-Metric-Induced Nonlinear Transport in the Layered Antiferromagnet CrSBr

**DOI:** 10.1021/acs.nanolett.5c00195

**Published:** 2025-05-27

**Authors:** Kamal Das, Yufei Zhao, Binghai Yan

**Affiliations:** † Department of Condensed Matter Physics, 34976 Weizmann Institute of Science, Rehovot 7610001, Israel; ‡ Department of Physics, The Pennsylvania State University, University Park, Pennsylvania 16802, United States

**Keywords:** van der Waals antiferromagnet, CrSBr, topological nodal line, nonlinear transport, quantum metric, surface-sensitive transport

## Abstract

The van der Waals (vdW) antiferromagnet CrSBr has recently garnered significant attention due to its air stability, high magnetic transition temperature, and semiconducting properties. We investigate its nonlinear transport properties and identify a quantum-metric-dipole (QMD)-induced nonlinear anomalous Hall effect and nonlinear longitudinal resistivity, which switch signs upon reversing the Néel vector. The significant QMD originates from Dirac nodal lines near the conduction band edge within the experimentally achievable doping range. Knowing the weak interlayer coupling, it is unexpected that the nonlinear conductivities do not scale with the sample thickness but are dominantly contributed by surface layers. In the electron-doped region, the top layer dominates the response, while the top three layers contribute the most in the hole-doped region. Our results establish topological nodal lines as a guiding principle to design high-performance nonlinear quantum materials, and we suggest that surface-sensitive transport devices will provide new avenues for nonlinear electronic applications.

## Graphical abstract

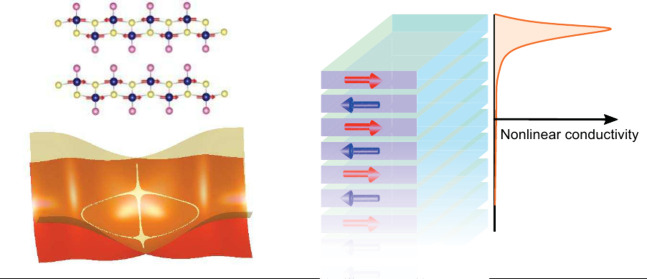

Magnetism in two-dimensional (2D) van der Waals (vdW) materials has recently gained intensive attention, challenging the long-held belief that intrinsic magnetic order could not be sustained in two dimensions due to enhanced thermal fluctuations.^1–3^ Although several 2D ferromagnets (FMs) and antiferromagnets (AFMs) have been demonstrated,^4–11^ most suffer from low magnetic order temperatures or extreme air sensitivity, limiting their further studies and practical applications. In this context, CrSBr has emerged as a promising vdW AFM with a simple magnetic structure characterized by spins along the *b* axis (AFM-*b*), exhibiting a high Néel temperature of 132 K, substantial air stability, and semiconducting properties.^12–24^ Unlike most vdW magnets, CrSBr displays in-plane magnetization,^13–15^ strong charge-spin^13,14^ and magneto-optical coupling,^17,20,22^ quasi-one-dimensional (1D) behavior,^25,26^ and controllable magnetic ordering.^27–29^ Its stability down to a few layers^14,16^ enables enhanced control over its properties, making CrSBr a promising candidate for future spintronic applications.^30–32^

The layered AFM structure of CrSBr is similar to the known AFM topological insulator MnBi_2_Te_4_, which exhibits notable nonlinear transport phenomena in thin films.^33,34^ While nonlinear responses have predominantly been investigated in topological materials due to their inherent quantum geometric effects,^35–37^ we point out that generic topological features near the Fermi energy, such as band crossing or anticrossing, will generate substantial Berry curvature or quantum metric even in an ordinary material. Therefore, we are motivated to explore the topological characteristics in the band structure of CrSBr and study possible nonlinear transport phenomena when the chemical potential is tuned close to the topological region by doping.

Remarkably, the band structure of CrSBr exhibits Dirac nodal lines within the experimentally accessible energy range, at about 63 meV above the conduction band minimum (CBM) and 250 meV below the valence band maxima (VBM). These nodal lines generate a significantly large quantum geometry and lead to a strong nonlinear transport. The even number of layers breaks inversion symmetry ( P ) but preserves the spatial-time reversal symmetry ( PT ), displaying the quantum-metric-dipole (QMD)-induced nonlinear anomalous Hall effect (NLAHE) and nonlinear longitudinal resistance—an effect observed only in a few magnetic materials so far.^33,34,38,39^ Intriguingly, the nonlinear conductivities (NLCs) in thick films do not scale with the sample thickness but are dominated by the surface layers, different from the linear transport, because surface layers exhibit strong inversion breaking while the inner layers do not. The outermost layer primarily dominates the nonlinear response in the electron-doped region, and the three outermost layers dominate in the hole-doped region. Our findings demonstrate the topological nodal line as a recipe of large nonlinear responses and highlight that the transport properties of CrSBr can be effectively probed using surface-sensitive techniques, for example, by placing the contact on the surface.

We first introduce the band structure. Recent studies reported distinct band structures with different methods.^26,40,41^ Transport and optical measurements^25,26^ showed a quasi-1D nature of material properties but varied band gaps. Recent angle-resolved photoemission spectroscopy (ARPES) data in the paramagnetic^42,43^ and AFM phases^41,44,45^ revealed more information on the band structure. Studies^41,45^ reveal that the conduction bands exhibit quasi-1D character, while the valence bands exhibit a quasi-2D nature. Top valence bands are contributed by the Cr t_2g_ orbital, while the lower valence bands are predominated by Br/S p orbitals. Several valence bands are located close in energy at the top of the X point. These ARPES features were reproduced by GW and dynamical mean-field-theory calculations.^41,45^ Although it produces a small band gap, similar data can be captured on the generalized gradient approximation (GGA) of the density functional theory (DFT) using Hubbard *U* = 0 eV. We show the band structure of the bulk CrSBr in the AFM-*b* phase for *U* = 0 eV in Figure 1a. In contrast, GGA+*U* calculations show different features from ARPES, although it is widely used in the literature. For example, the GGA+*U* top valence bands at X are sparsely spaced and Cr t_2g_ orbitals are overhybridized with other orbitals, as shown in Figure 1b. In addition, we find that DFT (*U* = 0 eV) correctly reproduces the AFM phase as the ground state while DFT+*U* (*U* = 3 eV) shows the FM phase as the ground state in calculations, consistent with a recent work.^28^ For these reasons, we will adopt GGA (*U* = 0 eV) calculations in this study. See details in sections 1 and 2 of the Supporting Information (hereafter SI).

**Figure 1:**
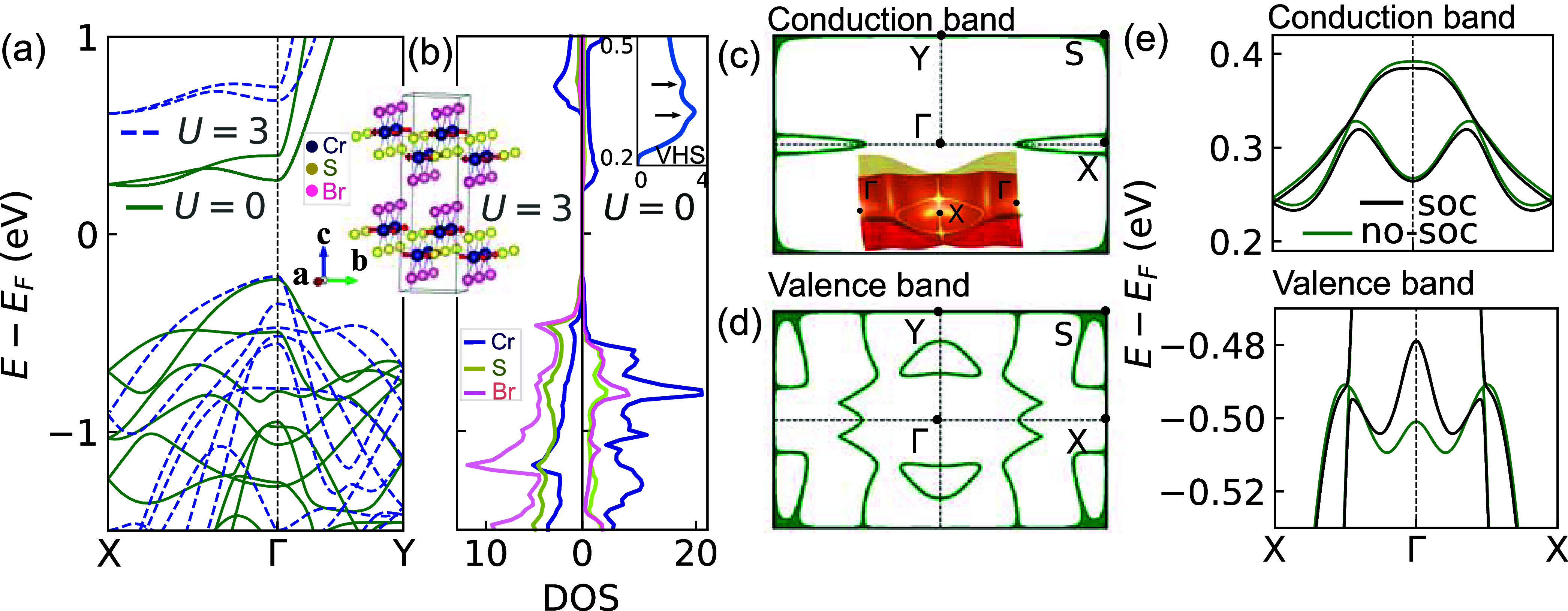
Crystal structure, electronic bands, and nodal lines of bulk CrSBr. (a) Band dispersion from GGA calculations without (solid green) and with (blue dashed) Hubbard *U* in the absence of SOC. The inset shows the AFM-*b* phase of bulk CrSBr. (b) Orbital projected density of states (DOS) for *U* = 3 eV (left panel) and *U* = 0 eV (right panel). The inset highlights the total DOSs near the CBMs for *U* = 0 eV, which shows the van Hove singularity (VHS). (c and d) Distribution of the Dirac nodal lines in the *k*_*x*_–*k*_*y*_ plane for the first conduction and valence bands, respectively. (e) Opening of the band gap in the presence of SOC in the conduction (top) and valence (bottom) bands.

We find a band crossing along the Γ–X path near the CBM while the valence band shows multiple crossings. The crossing points in the conduction bands extend to form an X-centric Dirac nodal ring, as shown in Figure 1c. In the valence band, the crossings extend into a more complex structure of Dirac nodal lines. Figure 1d shows the nodal lines for the first valence band. These Dirac-type band crossings are protected by the glide mirror symmetry *M̅*_*z*_ ≡ {*M*_*z*_|τ}, where $\tau =\left(\frac{1}{2},\;\frac{1}{2},\;0\right)$ is the translation, and the nodal lines lie within the glide mirror plane (*k*_*z*_ = 0). We mention that the precise location of the crossing and the resultant nodal lines are sensitive to the atomic positions and the correlation parameter *U*. For example, some studies report crossing near the CBM along the Γ–*Y* path,^26,44,46,47^ which is likely based on a relaxed lattice structure without including magnetism (see section 2 of the SI for details). Some calculations with *U* = 3 eV do not show any crossing near the CBM,^40,48,49^ while the band crossing along Γ–X is consistent with refs 17, 28, and 50. Future ARPES studies are called for the confirmation of these nodal lines.

This nodal line topology of the bulk is inherited from the monolayer symmetry. The monolayer and odd number of layers share the same glide mirror with the bulk, where the mirror plane lies inside the vdW layer and can be represented by the spin group $\left\{I||{\mathrm{M̅}}_{1z}\right\}$ , I , being the identity operation on spin. Therefore, they present the same Dirac crossing in the absence of spin–orbit coupling (SOC). In an even number of layers, the glide mirror plane shifts to the vdW gap, and the glide mirror *M̅*_2*z*_ connects the opposite spins of the adjacent layers. Consequently, it involves an extra spin rotation with the spin group operation $\left\{U\left(\pi \right)||{\mathrm{M̅}}_{2z}\right\}$ . Thus, the Dirac nodal lines are weakly gapped due to symmetry reduction even without SOC in the even number of layers. After SOC is included, all of these nodal lines are weakly gapped, regardless of the layer thickness (see section 3 of the SI for more details). Such a gap opening for the bulk is highlighted in Figure 1e in the conduction (top panel) and valence (bottom panel) bands along the X−Γ–X path. It is known that the gapped Dirac crossing (or anticrossing) commonly contributes a significant Berry curvature and leads to a large anomalous Hall effect^51^ or spin Hall effect^52^ in nodal line semimetals. As we will see below, the gapped Dirac crossings in CrSBr also generate a large quantum metric,^53^ which significantly enhances the nonlinear transport properties. Importantly, the nodal lines lie within the accessible energy range, in particular, on the conduction band side. In an early experiment,^13^ for example, CrSBr is electron-doped to the 2D carrier density *n*_2D_ ≈ 5 × 10^13^ cm^–2^, corresponding to a chemical potential of ≈70 meV above the CBM, which is close to the Dirac nodal line. Furthermore, in the charge-neutral pristine samples, doping of alkali atoms (e.g., Li, and K) can effectively shift the chemical potential, as seen in early experiments.^43,44,54^

With a comprehensive understanding of the band structure in place, we now move on to the nonlinear response of even-layer films of CrSBr. The response tensor is qualitatively determined by the space-time symmetries. Due to the combined PT symmetry, the Berry curvature dipole^55^ vanishes in this system. Hence, the NLCs within the relaxation time approximation^38,56,57^ are given by

$$\sigma_{i;jl}^{\mathrm{NLD}}=-\tau^{2}\frac{e^{3}}{\hbar^{3}}\underset{n}{\sum }\int_{k}f_{n}\partial_{i}\partial_{j}\partial_{l}\epsilon_{n}$$ 1a

$$\sigma_{i;jl}^{\mathrm{QMD}}=-\frac{e^{3}}{\hbar }\underset{n}{\sum }\int_{k}f_{n}\left[2\partial_{i}G_{jl}^{n}-\frac{1}{2}\left(\partial_{j}G_{il}^{n}+\partial_{l}G_{ij}^{n}\right)\right]$$ 1b

Here, ∂_*i*_ stands for ∂/∂*k*_*i*_, *f*_*n*_ for the Fermi function, ϵ_*n*_ for the energy, and $G_{ij}^{n}$ for the band normalized intraband quantum metric given by Gijn=∑p≠n2Re[⟨un|∂iH|up⟩⟨up|∂jH|un⟩]/(ϵn−ϵp)3 . Equation 1a represents the nonlinear Drude (NLD) contribution, which originates from the current-induced Fermi surface’s shift and is completely determined by the dispersive velocities of the wave packet. Contrary to this, eq 1b originates from the QMD defined as

$$\Lambda_{i;jl}^{n}=2\partial_{i}G_{jl}^{n}-\frac{1}{2}\left(\partial_{j}G_{il}^{n}+\partial_{l}G_{ij}^{n}\right)$$ 2

which is determined by the first derivative of the band-normalized quantum metric. Equation 1b has drawn attention recently because, unlike the Berry curvature, the quantum metric that represents the distance between two neighboring states in the momentum space hardly appears in transport, and this contribution is scattering-time-independent, being completely determined by the band structure (intrinsic). Furthermore, eq 1b has both longitudinal and transverse contributions. The longitudinal part causes a quantum geometric contribution to the unidirectional resistance, while the transverse part gives rise to an intrinsic NLAHE.^34,58^

In the ground-state AFM-*b*, even-layer films belong to the magnetic point group *m*′*mm* (magnetic space group *Pm*′*mn*), which includes the symmetries ${\mathrm{M̅}}_{y}\equiv \left\{M_{y}|\left(0,\;\frac{1}{2},\;0\right)\right\}$ , ${\mathrm{M̅}}_{x}T\equiv \left\{M_{x}|\left(\frac{1}{2},\;0,\;0\right)\right\}T$ , and *M̅*_*z*_. The in-plane NLCs are determined by *M̅*_*y*_ and ${\mathrm{M̅}}_{x}T$ symmetries, which impose the same constraints on the response tensors. Specifically, these symmetries forbid conductivities with an odd number of *y* indices: σ_*y*;*yy*_, σ_*y*;*xx*_, and σ_*x*;*yx*_(=σ_*x*;*xy*_). This can be understood by analyzing the real-space transformation of the current and electric fields under *M̅*_*y*_, where the current transforms as *j*_*y*_ → −*j*_*y*_ and *j*_*x*_ → *j*_*x*_ and the electric field as *E*_*y*_ → −*E*_*y*_ and *E*_*x*_ → *E*_*x*_. Consequently, only conductivities with an even number of *y*, namely, σ_*x*;*xx*_, σ_*x*;*yy*_, and σ_*y*;*yx*_ = σ_*y*;*xy*_, are allowed. Additionally, the mirror symmetry *M̅*_*y*_ forbids linear anomalous Hall conductivity. The response tensors for various spin orientations in the FM and AFM phases are highlighted in Table 1 (see section 6 of the SI for details). We present the variation of NLC σ_*x*;*yy*_ and σ_*x*;*xx*_ with the chemical potential for the bilayer system in the ground state in parts a and b of Figure 2, respectively. For scattering time τ = 0.01 ps, we find that QMD dominates the NLD contribution. Because the NLD contribution is more than an order smaller than the QMD conductivity, we will not discuss it further here but will provide a detailed analysis in section 6 of the SI. Interestingly, the conductivities peak only in a small energy window in the conduction band sector near μ = 0.28 eV (red dashed line). In the valence band sector, the NLC becomes significant inside the VBM after μ = −0.5 eV (blue dashed line). Notably, σ_*x*;*xx*_ is of the same order of magnitude compared to σ_*x*;*yy*_ on the valence band side but much smaller on the conduction band side; see the inset of Figure 2b. This is due to the quasi-1D and quasi-2D nature of the conduction and valence bands, respectively. We mention that the NLCs are calculated for a specific Néel configuration. When the spin orientations are reversed, i.e., up–down becomes down–up or vice versa, the NLCs change sign.^59^

**Table 1:** Magnetic Point Group (MPG) for Even- and Odd-Layer CrSBr for the Three Spin Orientations in the AFM and FM Phases^a^

spin axis	MPG	key symmetry	σ_*ij*_^AHE^	σ_*i*;*jl*_
Even Layers				
AFM-*b*	*m*′*mm*	*M̅*_*y*_	×	σ_*x*;*xx*_; σ_*x*;*yy*_; σ_*y*;*xy*_
AFM-*a*	*mm*′*m*	*M̅*_*x*_	×	σ_*y*;*xx*_; σ_*y*;*yy*_; σ_*x*;*xy*_
AFM-*c*	*m*′*m*′*m*′	${\mathrm{M̅}}_{y}T$ , ${\mathrm{M̅}}_{x}T$ , PT	×	×
FM-*b*	*m*′*mm*′	*M̅*_*y*_, P	×	×
FM-*a*	*mm*′*m*′	*M̅*_*x*_, P	×	×
FM-*c*	*m*′*m*′*m*	P	σ_*xy*_^AHE^	×
Odd Layers				
AFM/FM-*b*	*m*′*mm*′	*M̅*_*y*_, P	×	×
AFM/FM-*a*	*mm*′*m*′	*M̅*_*x*_, P	×	×
AFM/FM-*c*	*m*′*m*′*m*	P	σ_*xy*_^AHE^	×

a Listed are the allowed nonlinear transport (*J*_*i*_ = σ_*i*;*jl*_ *E*_*j*_ *E*_*l*_ with σ_*i*;*jl*_ = σ_*i*;*lj*_) and linear anomalous Hall (*J*_*i*_ = σ_*ij*_^AHE^ *E*_*j*_ with σ_*ij*_^AHE^ = −σ_*ji*_^AHE^) coefficients, where *i*, *j*, *l* ∈ *x*, *y*. The key symmetries that prohibit response coefficients are highlighted.

**Figure 2:**
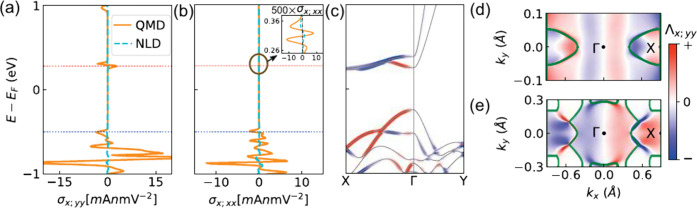
Nonlinear charge responses in bilayer CrSBr: (a) nonlinear transverse conductivity σ_*x*;*yy*_ and (b) longitudinal conductivity σ_*x*;*xx*_ for bilayer CrSBr in the AFM-*b* phase. The orange solid and cyan dashed lines are the contributions from the QMD and NLD, respectively. The inset in part b shows the contribution near the CBMs. (c) Band dispersion weighted by the QMD for the σ_*x*;*yy*_ conductivity. (d and e) Momentum space distribution of the QMD for the first conduction and valence bands. The high-concentration region follows the nodal line shown by the green lines.

The nodal lines play a pivotal role in shaping the NLCs. Specifically, the peaks and sign changes near μ = 0.25–0.35 eV and the onset of a significant response after μ = −0.5 eV are directly related to the position of the nodal lines, as demonstrated below for σ_*x*;*yy*_^QMD^. The QMD-weighted band dispersion in Figure 2c shows that all regions in the chemical potential where there is a peak in NLC correspond to some band crossing and the resultant large QMD. This behavior is further elucidated by the momentum-resolved QMD in the 2D Brillouin zone. The high intensity of QMD in Figure 2d, which roughly follows the nodal line structure shown by the green line, causes the peak in the NLC in the range of μ = 0.28–0.35 eV. In the valence band sector also, the large QMD follows the nodal line, as shown in Figure 2e. A relatively large contribution appears after μ = −0.5 eV compared to μ = −0.4 eV as the Fermi contour intersects the nodal lines with a high density of QMD, as illustrated in Figure 3f of the SI. This behavior is also associated with the orbital weight of magnetic Cr atoms, which is small for the valence band top but increases significantly below μ = −0.5 eV. Because magnetic states primarily govern the NLCs, the contribution remains small near the VBM. This makes detection of the topological nodal line and the resulting nonlinear transport more feasible to measure on the conduction band side. In Figure 3a, we have depicted the energy range of the nodal line (63–87 meV, red shaded region) and region of significant response (blue shaded) along with the experimentally reported chemical potential dashed green^13^ lines. Notably, the region of significant response extends beyond the nodal line region as SOC opens a slight gap, and, consequently, the QMD spreads below and above the gap. The envelope-like profile of the nonlinear response centered around the nodal line region is a characteristic feature of quantum-geometry-driven properties in both gapped and gapless systems, reflecting their underlying topological origin.

**Figure 3:**
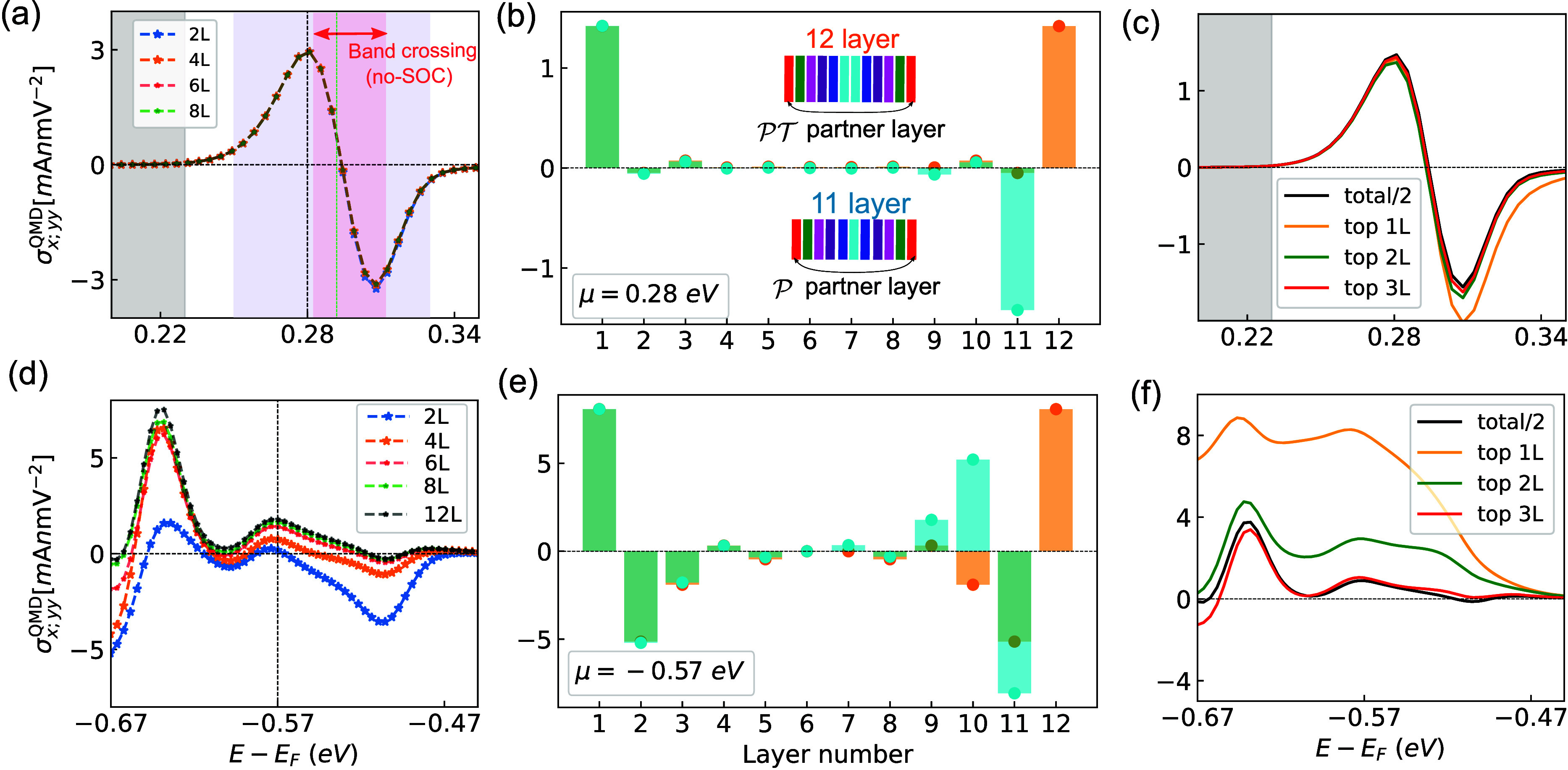
Surface-dominated nonlinear transport in thick films: The variation of the QMD contribution with the chemical potential for films with different thicknesses in the conduction band sector (a) and valence band sector (d). The gray shaded region shows the band gap. The red and blue shaded regions in part a show the nodal line energy range and significant conductivity region. The green dashed line shows the experimental chemical potential.^13^ The layer projection of the NLC for 12L (orange) and 11L (cyan) thick films at μ = 0.28 eV (b) and at μ = −0.57 eV (e). In the schematic of slabs, the same colored layers are a parity-time reversal partner in the 12L film and inversion partners in 11L. The NLC from the 1L, 2L, and 3L layers near the surface is compared with half of the total contribution (black) in the conduction (c) and valence (f) bands.

The optical and transport properties show a strong 2D nature with weakly coupled layers for CrSBr in optical and transport measurements.^21,26,60,61^ However, NLCs distribute nonuniformly among layers in a thin film. Here, we resolve the layer-by-layer contribution to NLC by using thick films as examples. In Figure 3a, we show the QMD-induced NLC from 2L–8L in the conduction band sector near μ = 0.25–0.35 eV. Intriguingly, the NLC does not scale with the layer number and is almost the same for all. This is related to the fact that the surface layer has a dominant contribution to NLC. Furthermore, we plot the layer projection of the QMD-induced NLC at μ = 0.28 eV for 12L- and 11L-thick films in Figure 3b. For both films, the inner layer contributes little, and most of the contribution arises from the outermost layer. Notably, the layers from the top and bottom sides have the exact same contributions for the 12L-thick film because they are PT partners of each other. For the 11L-thick film, the outer layers have opposite contributions because they are connected by P symmetry, leading to a vanishing total response. In Figure 3c, we compare half of the total contribution (black line) of the 12L film with the contribution from the top 1L (orange), 2L (green), and 3L (red). It is evident that the surface layers completely determine the total response of the thick film.

A similar layer projection analysis for the valence bands is shown in Figure 3d−f. The NLCs for 2L–8L and 12L are presented in Figure 3d. As observed in the conduction bands, the NLC does not scale with the number of layers. However, unlike the conduction bands, the valence band response requires more layers to reach saturation, which occurs after 8L. The higher number of layers required for saturation in the valence bands can be attributed to the stronger interlayer coupling through the atomic orbital dominant in these bands. The interlayer coupling occurs primarily through Br p orbitals^28^ because the Cr–S layers are sandwiched between Br layers. The Br p orbitals contribute significantly in the valence band top, while their contribution at the conduction band bottom is vanishingly small. Consequently, interlayer coupling is much weaker in these conduction bands than in the valence bands. The layer projected contribution at μ = −0.57 eV is shown in Figure 3e. Here, we note that only the outermost layer is insufficient to describe the overall transport, and one needs to consider the outer three layers. The corresponding chemical potential dependence of the outermost layer’s contribution is highlighted in Figure 3f, implying that the outermost three layers determine the total response.

The reason for the surface layer domination can be attributed to the symmetry. Each individual layer of CrSBr has an inversion symmetry. Due to this, layers that are deep inside the slab do not experience inversion breaking. However, the outermost layers feel the inversion breaking due to surface termination and hence make a dominant contribution to the nonlinear transport. We should point out that surface nonlinear Hall effects were reported for topological materials,^62,63^ where topological surface states lead to Berry curvature-related extrinsic nonlinear responses. Different from them, the CrSBr film exhibits no surface state, and every layer conducts electrons in ordinary transport. Therefore, it becomes nontrivial that the surface layer becomes dominant in nonlinear transport. In addition, we note that a surface contribution similar to that of the nonlinear optical effect was proposed in another layered AFM CrI_3_,^64^ in which the interband quantum metric representing the dipole transition between bands was discussed. Our studies focus on the topological nodal-line-induced intrinsic nonlinear transport, despite that extrinsic effects may also exist in experiments.^65–67^

In conclusion, the nonlinear transport is predominantly surface-driven and insensitive to the film thickness in CrSBr. This behavior arises from the surface inversion breaking and local single layer inversion symmetry, which is even independent of the underlying magnetic order (e.g., AFM or FM). Our findings go beyond the expected surface transport of axion insulators or topological insulators in which topological surface states constitute the Fermi surface. Our results indicate that surface-sensitive measurements, where contacts are placed on a single surface of a film, as shown in Figure 4, can effectively probe the nonlinear responses and quantum geometry. The sample thickness can be less sensitive in the device fabrication compared to ordinary thin-film devices.^33,34^ Our calculations indicate that the electron-doped sample will exhibit nonlinear responses much stronger than those of the hole-doped case. While CrSBr serves as the material platform in our study, the core findings—the surface-dominant quantum geometry and its origin in topological band structure—provide a general guideline and are broadly applicable to other materials. Our work paves a path to designing surface-sensitive nonlinear devices for applications in energy harvesting, photodetectors, and spintronics.

**Figure 4:**
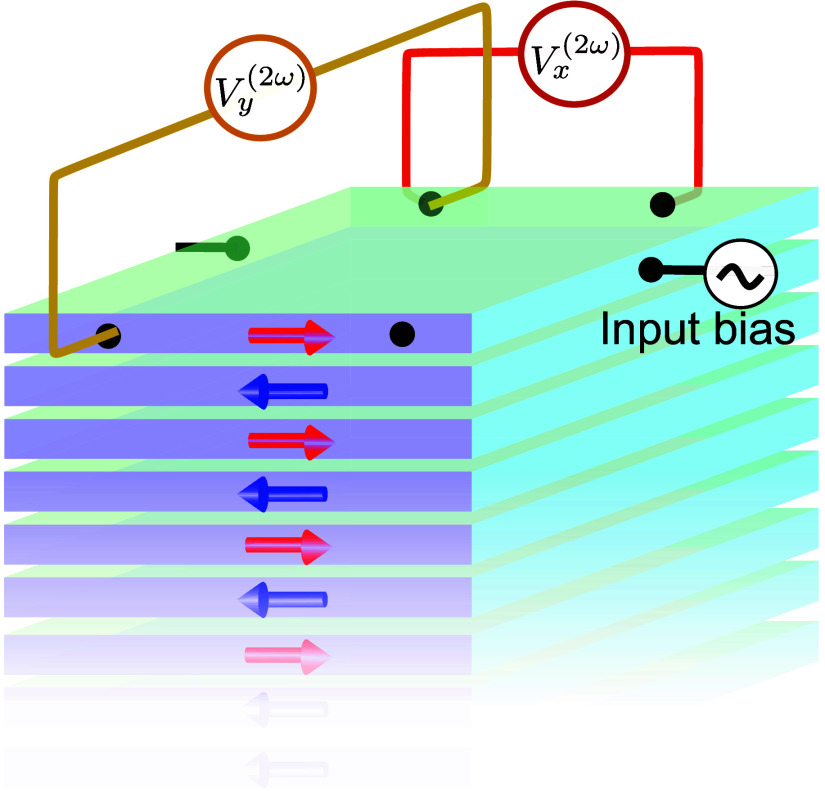
Schematic of the surface-sensitive nonlinear electronic device: Nonlinear transport through the source and drain grown on the surface. The yellow channel represents Hall effects and the red channel represents longitudinal nonlinear transport.

## Supplementary Material



## Notes

Methods of DFT calculations, effect of Hubbard *U* and relaxation on the band structure, symmetry protection of nodal lines in the absence of SOC, methods for slab calculation from bulk Hamiltonian, methods of NLC calculations, carrier concentration and energy contours in the Brillouin zone, and methods of layer-resolved calculation (PDF)
