# Supplementary material for: Surface-Dominated Quantum-Metric-Induced Nonlinear Transport in the Layered Antiferromagnet CrSBr
Source: Nano Lett. 2025 May 27;25(23):9189–96. doi: 10.1021/acs.nanolett.5c00195 (PMC12164527; doi:10.1021/acs.nanolett.5c00195)
Supplement: Supplementary file 1 [file nl5c00195_si_001.pdf]

Supporting Information: Surface-Dominated Quantum Metric-Induced Nonlinear Transport in the Layered Antiferromagnet CrSBr

Kamal Das[1],Yufei Zhao[1], and Binghai Yan[1,2*]

[1] *Department of Condensed Matter Physics, Weizmann Institute of Science, Rehovot 7610001, Israel*

[2] *Department of Physics, The Pennsylvania State University, University Park, Pennsylvania 16802, USA*

E-mail: binghai.yan@weizmann.ac.il

1 Methods of DFT calculations

The first-principle calculation is performed by Vienna Ab initio Simulation Package (VASP) with projector-augmented wave pseudopotentials[1,2]. For the AFM phase, we considered the experimentally realized lattice parameters and atomic positions provided in Ref.[3] at 1.8 K. No further optimization of the lattice parameters was performed. We used the Perdew-Burke-Ernzerhof (PBE) implementation of the generalized gradient approximation (GGA) for the exchange-correlation[4]. To consider the strong correlation of Cr-$3d$ orbitals, the Dudarev type GGA+U method has been used[5]. The plane wave energy cut-off is considered to be 400 eV and electronic energy optimization is down up to 1 μeV. We used Grimme's semiempirical correction method, DFT-D3 (IVDW=11) to include the vdW interaction of the system[6]. The self-consistent calculation are done using $12 \times 8 \times 4$ Γ-centered grid which gives the magnetic moment per Cr atom 3.181 $\mu_{\rm B}$ for $U = 3$ eV and 2.958 $\mu_{\rm B}$

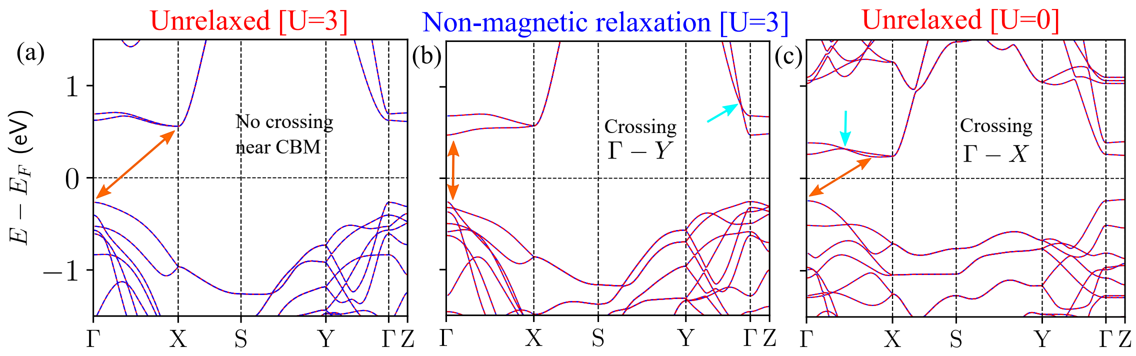

Figure 1: **Three types of band dispersion in AFM-b phase**. (a) Band structure obtained from experimental lattice parameters at $U = 3$ eV which shows no crossing near the CBM. The band gap is indirect. (b) Band structure obtained after nonmagnetic relaxation at $U = 3$ eV. It shows a direct band gap and the band crossing appears along the $\Gamma - Y$ path in the electron-doped region. (c) The band structure obtained from the experimental lattice parameters at $U = 0$ eV. It shows an indirect band gap and band crossing appears along $\Gamma - X$ path in the electron-doped region.

for $U = 0$ eV.

2 Effect of Hubbard U and relaxation on band structure

In this section, we discuss the effect of the electronic correlation parameter U and atomic relaxation on the band structure and compare the different band structures reported in the literature with our calculation. For our calculation of the bulk CrSBr, we consider the experimentally reported lattice parameters at 1.8 K in Ref.[3]. The unit cell parameters are given by $a = 3.5127$ Å, $b = 4.7458$ Å, and $c = 7.9131$ Å. The atomic positions of the Cr, S and Br atoms are $\{(0.5 \mp 0.25), (0.5 \pm 0.25), (0.5 \pm 0.1269)\}$, $\{(0.5 \pm 0.25), (0.5 \pm 0.25), (0.5 \mp 0.0739)\}$ and $\{(0.5 \pm 0.25), (0.5 \pm 0.25), (0.5 \pm 0.3535)\}$, respectively. For the antiferromagnet (AFM) phase, we double the cell along the c-axis.

Three distinct types of ground state band structures reported in the literature are shown in Fig. 1. The first, shown in Fig. 1(a) corresponds to band structure with no band crossing near the conduction band minima (CBM). This band structure is obtained using the experimental lattice parameters and the widely used electronic correlation parameter $U = 3$ eV. The band gap is found to be indirect. The band structure aligns poorly with the ARPES measurement as bands near the valence band

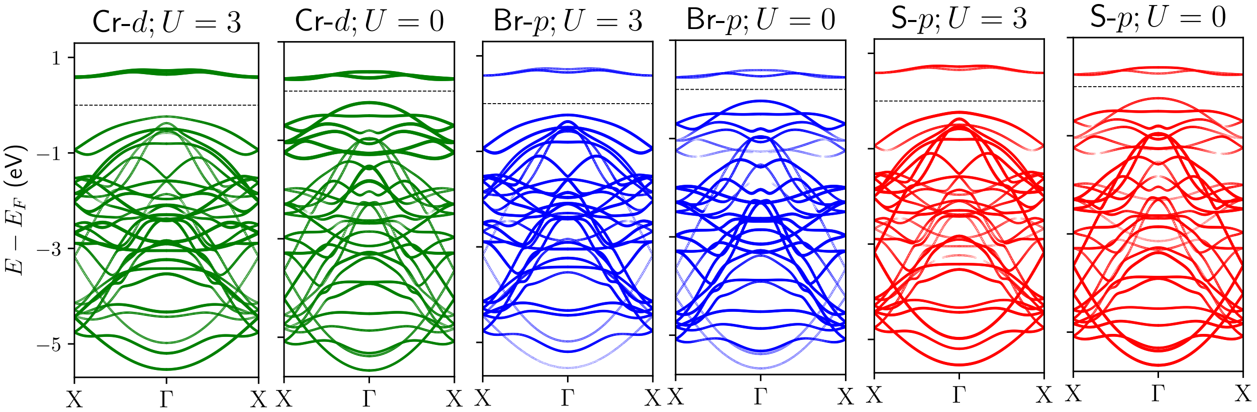

Figure 2: **Orbital projected bands for $U = 3$ eV and $U = 0$ eV:** The green colored lines show the projection on Cr-d orbitals. The blue color lines show the projection of the Br-p orbital and the red colored lines show the S$-p$ orbital weight.

maxima (VBM) are sparsely spaced near high symmetry point X.

The second reported band structure shown in Fig. 1(b) features band crossing along the $\Gamma - Y$ path[7–9] and exhibits a direct band gap unlike the previous case. We obtain this band structure after relaxing the atomic positions without incorporating magnetic moments in the unit cell and considering $U = 3$ eV. With this relaxation, the band structure bands deviate from the ARPES measurement. Importantly, for this and the previous case, a ferromagnetic (FM) ground state is preferred over the AFM phase differing from the experimental observations.

The third band structure shown in Fig. 1(c) shows band crossing along the $\Gamma - X$ path and obtained for experimental lattice parameters and $U = 0$ eV. The $U = 0$ eV electronic structure aligns most closely with the experimental findings among the three band structures. As detailed in the main text, it successfully reproduces the band features observed in recent ARPES measurements. Furthermore, for $U = 0$ eV, the AFM gound state is preferred with 0.4 meV energy per AFM unit cell over the FM phase.

What follows, we discuss the difference in the orbital weights in the bands for $U = 0$ eV and $U = 3$ eV shown in Fig. 2. We show the orbital projections along the $X - \Gamma - X$ path. The green, blue, and red represent the Cr-d, Br-p and S-p orbitals respectively. Notably, the Cr-d orbitals are more concentrated near the band extrema for $U = 0$ eV than $U = 3$ eV. For $U = 3$ eV the Cr-t_{2g} orbitals

Table 1: Symmetry operations and band crossing. In the absence of SOC, the spin group symmetry is shown, where the first and second sections represent spin operation and lattice transformation. In the presence of SOC, the ordinary symmetry is adopted where $\mathcal{T}$ is the time-reversal. $\overline{M}_{1z}$ is when the mirror plane is inside the layer and $\overline{M}_{2z}$ when the mirror plane lies in between two layers. The protected and gapped band crossings in the mirror plane are indicated for each cases.

System	Symmetry operation (no-SOC)	Band crossing	Symmetry operation (SOC)	Band crossing
Monolayer	$\{\mathcal{I} \parallel \overline{M}_{1z}\}$	✓	$\overline{M}_{1z}\mathcal{T}$	$\times$
Bilayer	$\{\mathcal{U}(\pi) \parallel \overline{M}_{2z}\}$	$\times$	$\overline{M}_{2z}$	$\times$
Bulk	$\{\mathcal{I} \parallel \overline{M}_{1z}\}$ and $\{\mathcal{U}(\pi) \parallel \overline{M}_{2z}\}$	✓	$\overline{M}_{1z}\mathcal{T}$ and $\overline{M}_{2z}$	$\times$

go well inside the valence band which poorly alignss with the ARPES findings. Furthermore, for $U = 3$ eV Br/S-p orbitals are significantly hybridized with the Cr-d orbitals, which is not the case with $U = 0$ eV.

3 Symmetry protection of nodal lines in the absence of SOC

The band crossing features discussed in the previous section is symmetry-protected. Here, we discuss the details of the symmetry protection. The overall scenario for the symmetry protection of the band crossing is summarized in Table. 1. We stress that the symmetry guarantees band-crossing protection only in the absence SOC for monolayer and bulk while the band gap opens by introducing SOC.

(i) Monolayer. In the absence of SOC, we use the spin group symmetry (see Table. 1). The $\overline{M}_{1z} \equiv \{M_{1z}|(\frac{1}{2}, \frac{1}{2}, 0)\}$ is the glide mirror plane lying inside the layer, and the spin remains unchanged. In the spin group, it can be represented by $\{\mathcal{I} \parallel \overline{M}_{1z}\}$ where the left and right sides represent operations on spin and space, respectively. In this case, $\{\mathcal{I} \parallel \overline{M}_{1z}\}$ can protect the nodal line inside the mirror plane. In the presence of SOC the nodal lines open a gap because the spin group symmetry is broken.

(ii) Bulk. Because bulk also has the same glide mirror $\{\mathcal{I} \parallel \overline{M}_{1z}\}$, it also has protected nodal lines

in the absence of SOC. In the presence of SOC, spin group symmetry is broken, and hence a slight band gap opens.

(iii) Bilayer. There is a different glide mirror $\overline{M}_{2z} \equiv \{M_{2z}|(\frac{1}{2}, \frac{1}{2}, 0)\}$ with the mirror plane between two layers to connect opposite spin sublattices. Unlike the monolayer, opposite spins between two layers add an additional operation of spin-flip in the spin group, i.e., $\{\mathcal{U}(\pi) \parallel \overline{M}_{2z}\}$. This additional spin flip reduces the symmetry of the system and generate a band gap even in the absence of SOC. Then this gap is further opened after including SOC. Although the $\overline{M}_{2z}$ symmetry get restored in the presence of SOC, this symmetry is not enough to guarantee a fourfold degeneracy in the spin degenerate bands of the AFM.

4 Methods for slab calculation from bulk Hamiltonian

The Wannier tight-binding model was constructed to calculate the nonlinear responses using the VASP2WANNIER90 code[10]. Following the *ab initio* orbital projections, we considered the Cr-d, S-p, and Br-p orbitals to construct the Wannier tight-binding Hamiltonian. The tight-binding parameters were further symmetrized by the WannierTools package[11]. From this tight-binding model, the density of states are calculated using $200 \times 200 \times 200$ $\boldsymbol{k}$-grid with Gaussian broadening 10 meV, and the nodal lines are obtained in 1400×1400 $\boldsymbol{k}$-grid with an energy gap smaller than 1 meV in Fig. 1 of the main text.

The band structure and different responses of the even and odd layers were calculated using slab models. Finite-size slabs of different numbers of layers were constructed from the bulk Wannier Hamiltonian. The truncation is done along the c-axis. Periodicity is maintained in the x and y direction, consequently, k_x/k_y is the good quantum number. The slab Hamiltonian for N layers in

the 2D momentum space defined by $\boldsymbol{k}_{\|}(k_x, ky)$ can be written as

$$\mathcal{H}^{slab}_{mn}(\boldsymbol{k}_{\|}) = \begin{pmatrix} H_{mn,11}(\boldsymbol{k}_{\|}) & H_{mn,12}(\boldsymbol{k}_{\|}) & \cdots & H_{mn,1N}(\boldsymbol{k}_{\|}) \\ H_{mn,21}(\boldsymbol{k}_{\|}) & H_{mn,22}(\boldsymbol{k}_{\|}) & \cdots & H_{mn,2N}(\boldsymbol{k}_{\|}) \\ \vdots & \vdots & \vdots & \vdots \\ H_{mn,N1}(\boldsymbol{k}_{\|}) & H_{mn,N2}(\boldsymbol{k}_{\|}) & \cdots & H_{mn,NN}(\boldsymbol{k}_{\|}) \end{pmatrix} . \quad (1)$$

The elements of the matrix are obtained as

$$H_{mn,ij}(\boldsymbol{k}_{\|}) = \sum_{\{\boldsymbol{R}_{\|},(1-\delta_{ij})\boldsymbol{R}_{\perp}\}} e^{i\boldsymbol{k}_{\|}\cdot\boldsymbol{R}} H_{mn}(\boldsymbol{R}) . \quad (2)$$

By diagonalizing the Hamiltonian given in Eq. (1) we obtained the energy bands of the slab system. We have shown the band structures for monolayer, bilayer, and 12L in Fig. 3(a)-(c) for antiferromagnetically coupled layers. We have checked that the band dispersion for slabs from our codes are consistent with slab bands from the WannierTools package.

Notably, the band structure does not change much with the increase of layer numbers (thickness), particularly in the electron-doped region. However, the bands in the valence band side modify a bit more compared to the conduction bands. This can be explained as follows. The bands near the VBM are dominated by the Cr-t_{2g} while the conduction band side is dominated by the Cr-e_g orbitals. In the antiferromagnetic phase, the magnetic Cr atoms in the adjacent layers are antiferromagnetically coupled through the Br-p orbitals as the Cr-S layers are sandwiched between the Br layer. We find that the orbital contribution of Br-p is much weaker in the electron-doped side compared to the valence band sector. Due to this, the inter-layer coupling is relatively stronger in the valence band side.

The nonlinear transport properties are also calculated following the slab Hamiltonian. For calculating the velocity, required for different nonlinear conductivities we take the momentum derivative of each element of the Hamiltonian given in Eq. (1).

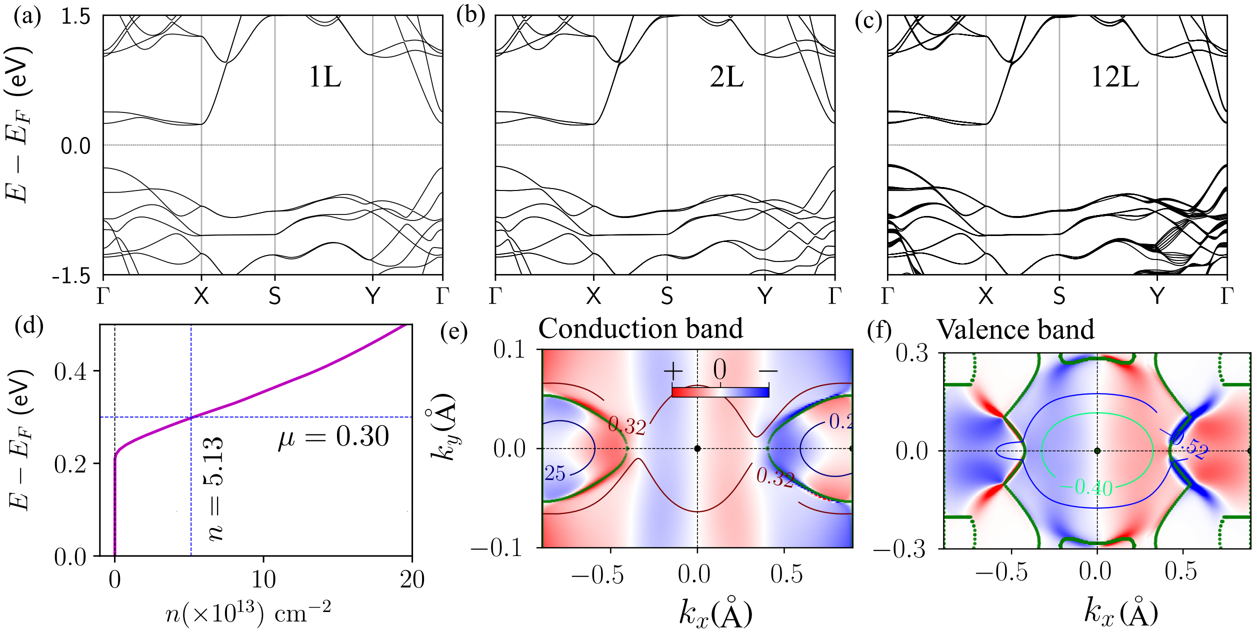

Figure 3: **Band dispersion, carrier concentration and energy contours**: (a)-(c) Band dispersion for monolayer, bilayer and 12L CrSBr films. The band structure does not change much as the number of layers increases, particularly in the conduction band sector. (d) Variation of the carrier concentration with chemical potential for monolayer. The blue dashed lines mark the experimental observed carrier concentration. (e)-(f) The distribution of the quantum metric dipole along with the equi-energy contours for the first conduction and valence band.

5 Carrier concentration and energy contours in the Brillouin Zone

In Fig. 3(d) we present the carrier concentration as a function of chemical potential for monolayer CrSBr. The vertical blue dashed line marks the previously reported carrier concentration while the corresponding energy for this carrier concentration $\mu = 0.30$ eV is highlighted by blue dashed horizontal line. Importantly, this energy level is very close to the band-crossing energy level.

In the main text, we have attributed the large nonlinear conductivities (NLCs) near the band crossing to the large quantum metric dipole (QMD). Here, we elaborate on that with the help of energy contours. In Fig. 3(e) we show the equi-energy contour in the 2D BZ along with the QMD. As the chemical potential shifts from $\mu = 0.25$ eV to $\mu = 0.32$ eV the Fermi surface traverses the region of high QMD concentration. The energy contours in the valence band side are shown

in Fig. 3(f). Here, the relatively large contribution at $\mu = -0.52$ eV compared to $\mu = -0.40$ eV is understood as the Fermi contour crosses the region of high density of QMD for the former chemical potential.

6 Methods of nonlinear conductivities calculations

The quantum metric dipole contribution appears from the band energy normalized quantum metric which is related to more general quantum geometric tensor

$$\mathcal{Q}_{jl}^{np} = \frac{\langle u_n(\boldsymbol{k})|\partial_j \mathcal{H}(\boldsymbol{k})|u_p(\boldsymbol{k})\rangle\langle u_p(\boldsymbol{k})|\partial_l \mathcal{H}(\boldsymbol{k})|u_n(\boldsymbol{k})\rangle}{(\epsilon_n - \epsilon_p)^2} \ . \tag{3}$$

Here, $|u_n(\boldsymbol{k})\rangle$ is the periodic part of the Bloch Hamiltonian $\mathcal{H}(\boldsymbol{k})$. The band normalized quantum metric and Berry curvature are obtained from the real and imaginary part as

$$\mathcal{G}_{jl}^{n} = \sum_{p\neq n} \frac{2\mathrm{Re}[\mathcal{Q}_{jl}^{np}]}{\epsilon_n - \epsilon_p}; \quad \Omega_{jl}^{n} = -\sum_{p\neq n} 2\mathrm{Im}[\mathcal{Q}_{jl}^{np}] \ . \tag{4}$$

The energy difference in the denominator often causes problems of convergence particularly when the two bands cross each other. To remove this degeneracy problem, we have used the a broadening parameter $\eta = 10^{-2}$. For the nonlinear Drude (NLD) contribution, we have used a revised formula[12]

$$\sigma_{a;bc}^{\mathrm{NLD}}(\tau^2) = -\tau^2 \frac{e^3}{2} \sum_n \int [d\boldsymbol{k}] v_n^a v_n^b v_n^c \partial_\epsilon^2 f_n \ . \tag{5}$$

The advantage of using this formula is that we can avoid the double derivative of energy which is computationally expensive. Furthermore, the cyclic nature of the NLD conductivity becomes evident from the expression. For the nonlinear conductivities in Fig. 2 of the main text, we have considered temperature T $= 100$ K and a 300×300 $\boldsymbol{k}$-grid for the Brillouin zone integration. The magnitude of the nonlinear conductivities is well converged for this k-grid.

For NLD conductivity, we considered the scattering time $\tau = 0.01$ ps. In the following we justify this value. Considering the experimental value of linear transport along the b-axis $\sigma_{bb} \approx 40 \times 10^{-6}$ S [13], the effective mass $m_Y^e \approx 0.14 m_e$ [9] (with m_e as the free electron mass) and $n \approx 1 \times 10^{17} m^{-2}$ [14], we estimate scattering time from the relation between Drude conductivity σ, carrier concentration (n), scattering time (τ) and effective mass (m^*) given by $\sigma = \frac{ne^2\tau}{m^*}$ as $\tau_b \approx 2 \times 10^{-15}$. Similarly, considering the experimental value of transport along the a-axis $\sigma_{aa} \approx 1 \times 10^{-6}$ S [13], $m_X^e \approx 7 m_e$ [9] and $n \approx 1 \times 10^{17} m^{-2}$ [14], we estimate $\tau_a \approx 2 \times 10^{-15}$. Thus, we find $\tau \approx 0.001$ ps which is an order of magnitude smaller than the value considered in our work. The parameter τ estimates the relative strength of the extrinsic nonlinear Drude conductivity and intrinsic quantum metric dipole conductivity. A larger value indicate dominant NLD conductivity. In our paper, we have considered a larger value as an upper bound of the nonlinear Drude conductivity. However, with a more realistic value the actual Drude conductivity would be more smaller than what is estimated in our paper.

The crystalline symmetry-allowed NLC are obtained using the following rule from Neumann's principle [15,16]

$$\sigma_{i;jl}^{\mathrm{NLD/QMD}} = \pm R_{ii'} R_{jj'} R_{ll'} \sigma_{i';j'l'}^{\mathrm{NLD/QMD}} \,. \tag{6}$$

Using this relation, the allowed conductivities are summarized in Table. 1 of the main text for different magnetic point groups corresponding to the different spin orientations in the AFM and FM thin film phases. The simple way to understand symmetry analysis in the ground-state is discussed in the main text. Here, we discuss the rest of the phases. The AFM-a phase belongs to the point group $mm'm$ with symmetry $\overline{M}_x$, $\overline{M}_y\mathcal{T}$ and $\overline{M}_z$. The out-of-plane mirror symmetry does not play any role in determining the in-plane nonlinear conductivities, which are instead determined by in-plane symmetries. Both symmetries transform current as $j_y \to j_y$, $j_x \to -j_x$. Meanwhile $\overline{M}_x$ transforms the electric field as $E_x \to -E_x$ and $E_y \to E_y$ and $\overline{M}_y\mathcal{T}$ transform field as $E_y \to -E_y$ and $E_x \to E_x$. As a result $\sigma_{x;xx}, \sigma_{x;yy}$ and $\sigma_{y;yx} = \sigma_{y;xy}$ components are not allowed. Additionally, the mirror symmetry $\overline{M}_x$ also forbids linear anomalous Hall conductivity.

In the AFM-c phase $\overline{M}_x\mathcal{T}$ kills coefficients with an odd number of y and $\overline{M}_y\mathcal{T}$ kills responses with an odd number of x effectively making all the responses zero. The $\mathcal{PT}$ suppress the linear anomalous Hall conductivity. For rest of the phases, the inversion symmetry is present which forbids any quadratic nonlinear response.

7 Methods of layer resolved calculation

For layer-resolved transport calculation, we follow the approach proposed in Ref.[17,18]. The layer-resolved current is given by

$$\boldsymbol{j}(L) = \langle L|\hat{\boldsymbol{v}}\hat{\rho}|L\rangle \ , \tag{7}$$

where $|L\rangle$ represents the state vector for the layer L. Here, $\hat{\boldsymbol{v}}$ is the velocity operator and $\hat{\rho}$ represents the occupation. This definition equivalently can be written as

$$\boldsymbol{j}(L) = \sum_n \langle n|\hat{P}_L\hat{\boldsymbol{v}}\hat{\rho}|n\rangle \ , \tag{8}$$

where the layer operator is defined as $\hat{P}_L = |L\rangle\langle L|$. This can be proved by inserting complete sets as $\sum_{m,n,p}\langle L|m\rangle\langle m|\hat{\boldsymbol{v}}|p\rangle\langle p|\hat{\rho}|n\rangle\langle n|L\rangle$ in Eq. (7). Inserting complete sets in Eq. (8) leads to identical expressions. The use of a layer projection operator helps numerical implementation. For the calculation from the Wannier Hamiltonian, $\hat{P}_L = \sum_{l_i\in L}|\psi(l_i)\rangle\langle\psi(l_i)|$ where $|\psi(l_i)\rangle$ are the Wannier functions centered around atom l_i belonging to the layer L.

The nonlinear Drude conductivity effectively can be written to originate from the intra-band velocity $\langle n|\hat{\boldsymbol{v}}|n\rangle = v_n$ combined with the second-order distribution function $\langle n|\hat{\rho}^{(2)}|n\rangle = v_n^b v_n^c \partial_\epsilon^2 f_n$. Therefore, the layer-resolved contribution is obtained from the following expression

$$\sigma_{a;bc}^{\mathrm{NLD}}(L) = -\tau^2\frac{e^3}{2}\sum_n\int[d\boldsymbol{k}]\langle n|\hat{P}_L\hat{v}^a|n\rangle v_n^b v_n^c \partial_\epsilon^2 f_n \ . \tag{9}$$

Here, the $\langle n|\hat{P}_L\hat{v}^a|n\rangle$ layer projected velocity. The variation of the nonlinear Drude contribution

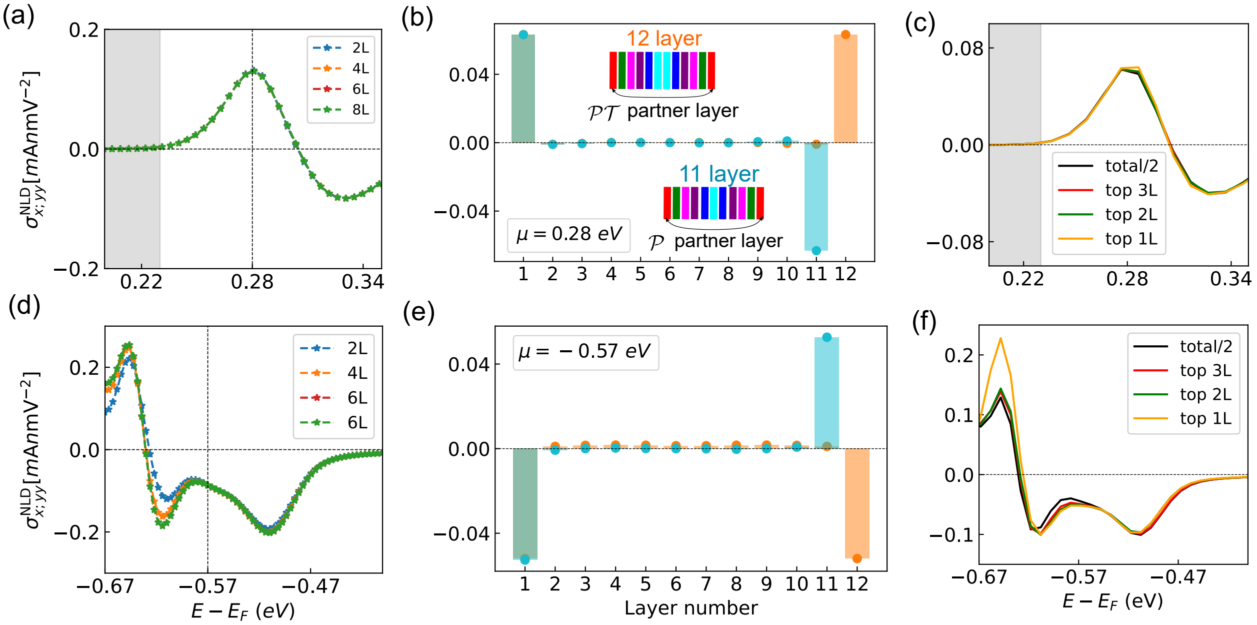

Figure 4: **Layer dependent study of the nonlinear Drude conductivity:** The variation of the nonlinear Drude contribution with chemical potential for films with different thickness in the conduction band sector (a) and valence band sector (d). The shaded region shows the band gap. The layer projection of the nonlinear conductivity for 12L (orange) and 11L (cyan) thick films at $\mu = 0.28$ eV (b) and at $\mu = -0.57$ eV (e). In the schematic of slabs, the same colored layers are a parity-time reversal partner in 12L film and inversion partners in 11L. The nonlinear conductivity from 1L, 2L, and 3L layers near the surface is compared with half of the total contribution (black) in the conduction band (c) and valence band (f).

with chemical potential for films with different thickness in the conduction band sector (a) and valence band sector (d). The shaded region shows the band gap. The layer projection of the nonlinear conductivity for 12L (orange) and 11L (cyan) thick films at $\mu = 0.28$ eV (b) and at $\mu = -0.57$ eV (e). In the schematic of slabs, the same colored layers are a parity-time reversal partner in 12L film and inversion partners in 11L. The nonlinear conductivity from 1L, 2L, and 3L layers near the surface is compared with half of the total contribution (black) in the conduction band (c) and valence band (f).

The Fermi surface form of the QMD conductivity is given by

$$\sigma^{\mathrm{QMD}}_{i;jl} = \frac{e^3}{\hbar} \sum_n \int_{\boldsymbol{k}} \left[2\mathcal{G}^n_{jl} \partial_i f_n - \frac{1}{2} \left(\mathcal{G}^n_{il} \partial_j f_n + \mathcal{G}^n_{ij} \partial_l f_n \right) \right]. \tag{10}$$

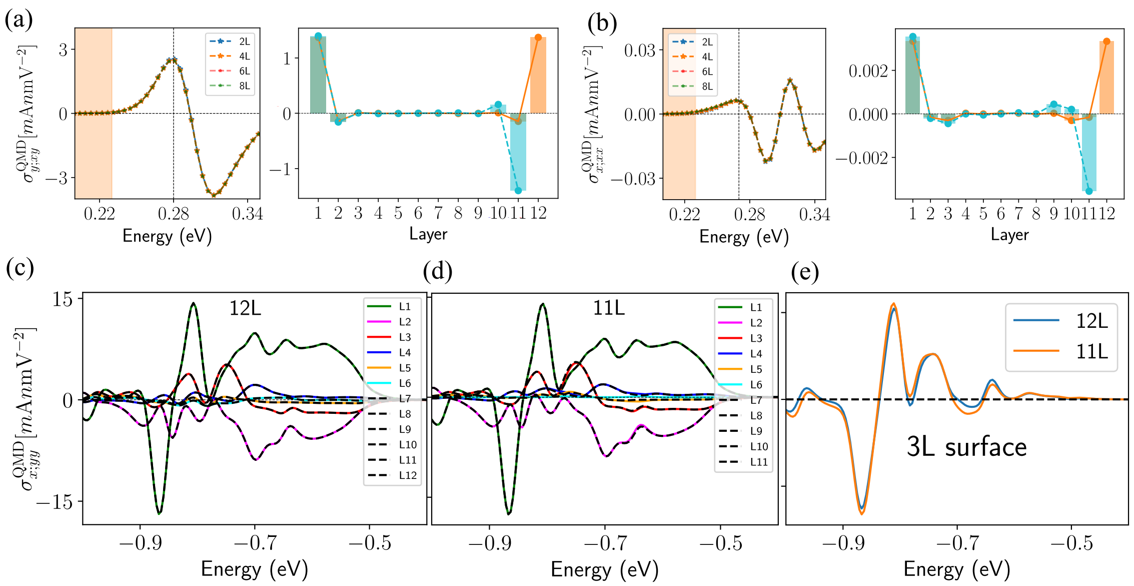

Figure 5: **Layer resolved nonlinear conductivity**: (a)-(b) [Left panels] The chemical potential dependence of the NLCs $\sigma^{\rm QMD}_{y;xy}$ and $\sigma^{\rm QMD}_{x;xx}$ for 2L-8L slab in the electron doped region. [Right panels] The layer projected contributions for 11L and 12L slab. (c) The chemical potential dependence of the layer resolved nonlinear conductivity for a 12 layer thick film. The dahed lines represent the contribution from the $\mathcal{PT}$ symmetry related layer. (d) The layer resolved nonlinear conductivity for a 11 layer system. Note that we have plotted the contribution from the $\mathcal{P}$ related layer with a negative sign using the dashed lines. (e) The outermost three layer contribution for 12L and 11L systems.

Although written in a single-band form, this contribution originates from interband coherence. Each term can be written in the multi-band form as

$$\sigma^{\rm QMD}_{i;ii} = \frac{e^3}{\hbar}\sum_n \int_{\boldsymbol{k}} \frac{2{\rm Re}[\langle n|\partial_i \mathcal{H}|m\rangle\langle m|\partial_i \mathcal{H}|n\rangle]}{(\epsilon_n - \epsilon_m)^3}\partial_i f_n. \tag{11}$$

This can be effectively considered as a product of the off-diagonal velocity $\langle n|\hat{\boldsymbol{v}}|m\rangle$ combined and the density matrix $\langle m|\hat{\rho}^{(2)}|n\rangle$. Therefore the layer-resolved contribution for the longitudinal component can be obtained from

$$\sigma^{\rm QMD}_{x;xx}(L) = \frac{e^3}{\hbar} 2{\rm Re}\sum_{n\neq m}\int_{\boldsymbol{k}} \langle n|\hat{P}_L \hat{v}_x|m\rangle\langle m|\hat{v}_x|n\rangle\partial_{k_x} f_n \ . \tag{12}$$

Similarly, the layer-resolved transverse quantum-metric dipole conductivity $\sigma^{\rm QMD}_{x;yy}$ can be calculated

using

$$\sigma^{\mathrm{QMD}}_{x;yy}(L) = \frac{e^3}{\hbar}\int_{\boldsymbol{k}} 2\mathrm{Re}\sum_{m\neq n} \tag{13}$$
$$\times \quad \frac{2\langle n|\partial_x\mathcal{H}|n\rangle\langle n|\hat{P}(L)\partial_y\mathcal{H}|m\rangle\langle m|\partial_y\mathcal{H}|n\rangle - \langle n|\partial_y\mathcal{H}|n\rangle\langle n|\hat{P}(L)\partial_x\mathcal{H}|m\rangle\langle m|\partial_y\mathcal{H}|n\rangle}{(\epsilon_n-\epsilon_m)^3}\frac{\partial f_n}{\partial \epsilon_n}\,.$$

The calculated layer-resolved contributions are shown in Fig. 5(c)-(e). The layer resolved contribution of the conductivities $\sigma^{\mathrm{QMD}}_{y;xy}$ and $\sigma^{\mathrm{QMD}}_{x;xx}$ are shown in Fig. 5(a)-(b) in the electron-doped region which shows the surface-dominated conductivity.

We performed several checks for the layer-resolved conductivity. Figure 5(c) shows the different layer contributions of $\sigma^{\mathrm{QMD}}_{x;yy}$ for 12L thick film. While different colors are used for the upper half of the thick film, black dashed lines have been used for the lower layers. All the $\mathcal{PT}$ partner layers have exactly the same contribution as expected. Additionally, it is checked that the sum of the layer contributions is the same as the 12L slab contribution. The outer layers, for instance, the green and pink lines show the maximum contribution implying that indeed the outermost layers have the most significant contribution. The same has been shown for 11L thick film in Fig. 5(d). The only difference is that to highlight that the $\mathcal{P}$ connected layers contribute exactly the opposite we have plotted the minus of the contribution from the lower set of the layer. Again the total from the layers is found to be zero as expected. We compare the contribution from the outermost three layers of 11L and 12L slab in Fig. 5(e) which shows that for thick films surface response for even and odd layer films are the same.

References

(1) Kresse, G.; Furthmüller, J. Efficient iterative schemes for ab initio total-energy calculations using a plane-wave basis set. *Phys. Rev. B* **1996**, *54*, 11169–11186.

(2) Blöchl, P. E. Projector augmented-wave method. *Phys. Rev. B* **1994**, *50*, 17953–17979.

(3) López-Paz, S. A.; Guguchia, Z.; Pomjakushin, V. Y.; Witteveen, C.; Cervellino, A.; Luetkens, H.; Casati, N.; Morpurgo, A. F.; von Rohr, F. O. Dynamic magnetic crossover at the origin of the hidden-order in van der Waals antiferromagnet CrSBr. *Nature Communications* **2022**, *13*, 4745.

(4) Perdew, J. P.; Burke, K.; Ernzerhof, M. Generalized Gradient Approximation Made Simple. *Phys. Rev. Lett.* **1996**, *77*, 3865–3868.

(5) Dudarev, S. L.; Botton, G. A.; Savrasov, S. Y.; Humphreys, C. J.; Sutton, A. P. Electron-energy-loss spectra and the structural stability of nickel oxide: An LSDA+U study. *Phys. Rev. B* **1998**, *57*, 1505–1509.

(6) Grimme, S.; Antony, J.; Ehrlich, S.; Krieg, H. A consistent and accurate ab initio parametrization of density functional dispersion correction (DFT-D) for the 94 elements H-Pu. *The Journal of Chemical Physics* **2010**, *132*, 154104.

(7) Yang, K.; Wang, G.; Liu, L.; Lu, D.; Wu, H. Triaxial magnetic anisotropy in the two-dimensional ferromagnetic semiconductor CrSBr. *Phys. Rev. B* **2021**, *104*, 144416.

(8) Bo, X.; Li, F.; Xu, X.; Wan, X.; Pu, Y. Calculated magnetic exchange interactions in the van der Waals layered magnet CrSBr. *New Journal of Physics* **2023**, *25*, 013026.

(9) Klein, J. et al. The Bulk van der Waals Layered Magnet CrSBr is a Quasi-1D Material. *ACS Nano* **2023**, *17*, 5316–5328, PMID: 36926838.

(10) Pizzi, G. et al. Wannier90 as a community code: new features and applications. *Journal of Physics: Condensed Matter* **2020**, *32*, 165902.

(11) Wu, Q.; Zhang, S.; Song, H.-F.; Troyer, M.; Soluyanov, A. A. WannierTools : An open-source software package for novel topological materials. *Computer Physics Communications* **2018**, *224*, 405 – 416.

(12) Železný, J.; Fang, Z.; Olejník, K.; Patchett, J.; Gerhard, F.; Gould, C.; Molenkamp, L. W.; Gomez-Olivella, C.; Zemen, J.; Tichý, T.; Jungwirth, T.; Ciccarelli, C. Unidirectional magnetoresistance and spin-orbit torque in NiMnSb. *Phys. Rev. B* **2021**, *104*, 054429.

(13) Wu, F.; Gutiérrez-Lezama, I.; López-Paz, S. A.; Gibertini, M.; Watanabe, K.; Taniguchi, T.; von Rohr, F. O.; Ubrig, N.; Morpurgo, A. F. Quasi-1D Electronic Transport in a 2D Magnetic Semiconductor. *Advanced Materials* **2022**, *34*, 2109759.

(14) Telford, E. J.; Dismukes, A. H.; Lee, K.; Cheng, M.; Wieteska, A.; Bartholomew, A. K.; Chen, Y.-S.; Xu, X.; Pasupathy, A. N.; Zhu, X.; Dean, C. R.; Roy, X. Layered Antiferromagnetism Induces Large Negative Magnetoresistance in the van der Waals Semiconductor CrSBr. *Advanced Materials* **2020**, *32*, 2003240.

(15) Gallego, S. V.; Etxebarria, J.; Elcoro, L.; Tasci, E. S.; Perez-Mato, J. M. Automatic calculation of symmetry-adapted tensors in magnetic and non-magnetic materials: a new tool of the Bilbao Crystallographic Server. *Acta Crystallographica Section A* **2019**, *75*, 438–447.

(16) Zhang, Z.-F.; Zhu, Z.-G.; Su, G. Symmetry dictionary on charge and spin nonlinear responses for all magnetic point groups with nontrivial topological nature. *National Science Review* **2023**, *10*, nwad104.

(17) Rauch, T. c. v.; Olsen, T.; Vanderbilt, D.; Souza, I. Geometric and nongeometric contributions to the surface anomalous Hall conductivity. *Phys. Rev. B* **2018**, *98*, 115108.

(18) Zhao, Y.; Jiang, Y.; Bae, H.; Das, K.; Li, Y.; Liu, C.-X.; Yan, B. Hybrid-order topology in unconventional magnets of Eu-based Zintl compounds with surface-dependent quantum geometry. *Phys. Rev. B* **2024**, *110*, 205111.
